# Supplementary material for: A Broad-Spectrum Monoclonal Antibody-Based Heterologous ic-ELISA for the Detection of Multiple Pyrethroids in Water, Milk, Celery, and Leek
Source: Foods. 2025 Feb 24;14(5):768. doi: 10.3390/foods14050768 (PMC11898949; doi:10.3390/foods14050768)
Supplement: Supplementary file 1 [file foods-14-00768-s001.zip › foods-3457936-supplementary.pdf]

A Broad-Spectrum Monoclonal Antibody-Based Heterologous ic-ELISA for the Detection of Multiple Pyrethroids in Water, Milk, Celery, and Leek

Table S1. Identify of subtype of mAb CL-CN/1D2

Subtype	IgG1	IgG2a	IgG2b	IgG3	IgA	IgM	Kappa	Lambda
OD_{450nm}	0.435	0.122	0.147	0.053	0.081	0.075	0.574	0.065

Note：$OD_{450nm} > 0.2$ was considered positive.

Table S2. Square matrix titration of mAb CL-CN/1D2

CL-CN/1D2(1:X [1])	B_1-DCC-BSA (1:X [1])						
	150	250	500	1000	2000	4000	8000
300	2.243	2.255	2.183	2.247	2.266	2.284	1.687
600	2.192	2.189	2.166	2.197	2.228	2.173	1.052
1200	2.197	2.19	2.167	2.226	2.224	**2.095**	0.914
2400	2.13	2.114	2.114	2.121	**2.069**	1.450	0.595
4800	2.008	1.989	1.923	1.957	1.720	1.085	0.459
9600	1.414	1.290	1.256	1.073	0.892	0.579	0.315
19200	0.636	0.638	0.571	0.535	0.466	0.353	0.339

Note：The conjugation ratios of B_1-DCC-BSA was13.4. [1]: "X" represents the dilution factor.

Table S3. Square matrix titration of mAb CL-CN/1D2

CL-CN/1D2(1:X [1])	B_2-DCC-BSA (1:X [1])						
	150	250	500	1000	2000	4000	8000
300	2.258	2.252	2.272	2.243	2.301	2.326	2.225
600	2.215	2.240	2.246	2.222	2.302	2.272	1.877
1200	2.211	2.262	2.260	2.249	2.300	**2.241**	1.514
2400	2.22	2.231	2.234	2.189	2.223	**1.988**	0.886
4800	2.17	2.166	2.126	2.058	**1.993**	1.414	0.573
9600	1.627	1.556	1.379	1.353	1.102	0.725	0.369
19200	0.536	0.709	0.540	0.450	0.496	0.351	0.270

Note：The conjugation ratios of B_2-DCC-BSA was 17.4. [1]: "X" represents the dilution factor.

Table S4. Square matrix titration of mAb CL-CN/1D2

CL-CN/1D2(1:X [1])	B_3-DCC-BSA (1:X [1])						
	24	48	97	194	388	1550	3100
225	2.504	2.455	2.498	2.519	2.535	2.505	2.502
450	2.464	2.450	2.445	2.468	2.502	2.491	2.404
900	2.435	2.429	2.441	2.438	2.466	2.424	2.412
1800	2.378	2.353	2.353	2.354	2.385	2.209	2.125
3600	2.254	2.280	2.255	2.209	2.102	**2.069**	1.842
7200	1.546	1.481	1.304	1.291	1.128	0.675	0.453
14400	0.396	0.415	0.385	0.362	0.378	0.321	0.307

Note：The conjugation ratios of B_3-DCC-BSA was15.3. [1]: "X" represents the dilution factor.

Table S5. Square matrix titration of mAb CL-CN/1D2

CL-CN/1D2(1:X [1])	B_4-DCC-BSA (1:X [1])						
	150	250	500	1000	2000	4000	8000
300	2.207	2.203	2.198	2.242	2.231	2.257	2.257
600	2.184	2.174	2.147	2.219	2.191	2.206	1.877
1200	2.188	2.170	2.138	2.205	2.201	2.134	1.533
2400	2.090	2.013	2.076	2.060	1.833	1.681	0.71
4800	1.861	1.879	1.861	1.849	1.592	1.224	0.508
9600	0.977	0.992	0.964	0.854	0.778	0.608	0.433
19200	0.394	0.539	0.422	0.406	0.393	0.352	0.309

Note：The conjugation ratios of B_4-DCC-BSA was16.0. [1]: "X" represents the dilution factor.

Table S6. Optimization of B-DCC-BSA concentration.

Coating antigen	Coating antigen (1:X [1])	CL-CN/1D2 (1:X [1])	“0” OD_{450nm}	IC_{50} (μg/L)
B_1-DCC-BSA	4000	1200	2.169	299.8
B_1-DCC-BSA	2000	2400	2.056	218.6
B_2-DCC-BSA	4000	1200	2.253	319.4
B_2-DCC-BSA	2000	2400	2.212	290.0
B_2-DCC-BSA	2000	4800	2.168	235.5
B_3-DCC-BSA	1550	3600	2.054	190.7
B_4-DCC-BSA	4000	1200	2.199	256.7
B_4-DCC-BSA	1000	2400	2.269	297.1

Note：The conjugation ratios of B_1-DCC-BSA, B_2-DCC-BSA, B_3-DCC-BSA, B_4-DCC-BSA were 13.4, 17.4, 15.3, 16.0, respectively. [1]: "X" represents the dilution factor.

Table S7. Optimization of mAb CL-CN/1D2 dilution

Coating antigen	Coating antigen (1:X [1])	CL-CN/1D2(1:X [1])	"0" OD_{450nm}	IC_{50}(μg/L)
		2000	1.899	217.0
		2200	1.805	228.8
B_1-DCC-BSA	2000	2400	1.813	197.2
		2600	1.457	196.7
		2800	1.569	202.6
		4400	2.189	228.1
		4600	2.188	281.1
B_2-DCC-BSA	2000	4800	2.18	229.8
		5000	2.155	168.8
		5200	2.013	219.1
		2800	2.286	183.2
		3200	2.286	170.3
B_3-DCC-BSA	1550	3600	2.281	161.8
		4000	2.208	168.6
		4400	2.208	181.5

Note：The conjugation ratios of B_1-DCC-BSA, B_2-DCC-BSA, B_3-DCC-BSA were 13.4, 17.4, 15.3, respectively. [1]: "X" represents the dilution factor.

Table S8. The LOD, LOQ, recoveries and CV (%) of the ic-ELISA for pyrethroids detection in lake water, milk, celery and leek samples.

Samples	Compounds	LOD (μg/kg)	LOQ (μg/kg)	Spiked level	Recovery±SD (%)	CV% (n=15)
Lake water	Cypermethrin	25.2	44.4	40, 80, 160	90.7 ± 5.0, 77.4± 9.1, 96.6 ± 14.1	9.2, 9.8, 6.1
	β-cypermethrin	24.4	36.6	40, 80, 160	90.5 ± 4.4, 93.2±9.4, 102.7 ± 9.0	12.1, 12.7, 5.5
	Cyfluthrin	36.0	54.7	60, 120, 240	95.5 ± 5.2, 101.8± 10.1,92.7 ± 16.8	11.0, 10.0, 9.0
	λ-cyhalothrin	43.2	55.8	50, 100, 200	92.7 ± 6.1, 103.0 ± 5.6, 96.5 ± 9.6	13.1, 5.4, 5.0
	β-cyfluthrin,	46.0	58.3	50, 100, 200	74.1 ± 6.2, 105.4±8.2, 100.1 ± 7.8	14.8, 7.7, 3.9
	Fenpropathrin	40.0	56.1	50, 100, 200	93.3 ± 6.2, 106.7±3.6, 98.6 ± 6.0	14.8, 3.4, 3.0
	Deltamethrin	25.2	44.4	75, 150, 300	89.8 ± 6.2, 102.3± 10.9, 93.2 ± 12.9	11.5, 8.9, 5.8
	Fenvalerate	45.7	75.1	75, 150, 300	91.0 ± 4.9, 81.9 ± 5.6, 86.7 ± 12.3	5.3, 6.7, 7.3
Milk	Cypermethrin	37.5	53.3	50(MRL), 100, 200	104.2 ± 8.8, 82.8±13.5, 88.3 ± 9.1	14.0, 13.6, 4.3
	β-cypermethrin	37.5	52.8	50(MRL), 100, 200	106.2 ± 7.9, 104.4± 5.0, 103.0 ± 10.1	12.4, 4.0, 4.1
	Cyfluthrin	59.0	87.0	80, 160, 320	96.6 ± 6.3, 96.2 ± 10.9, 84.7 ± 8.2	10.2, 8.8, 3.8
	λ-cyhalothrin	52.7	82.8	80, 160, 200(MRL), 320	83.9 ± 5.1, 100.5 ± 5.8, 97.9 ± 7.5, 96.8 ± 14.7	9.4, 4.5, 12.3, 5.9
	β-cyfluthrin,	57.4	80.6	80, 160, 320	79.4 ± 4.3, 102.6 ±5.8, 92.1 ± 7.3	12.3, 12.5, 6.7
	Fenpropathrin	52.0	81.0	80, 160, 320	66.4 ± 4.6, 92.5±11.9, 92.5 ± 13.1	10.8, 10.1, 5.5
	Deltamethrin	67.4	95.4	100, 200, 400	103.8 ± 9.7, 96.4±5.8, 96.3±12.1	10.5, 4.6, 5.1
	Fenvalerate	68.9	96.9	100(MRL), 200, 400	94.5 ± 8.6, 103.7 ± 5.5, 86.4 ± 10.1	12.3, 6.2, 8.5
Celery	Cypermethrin	68.5	97.2	100, 200, 400, 1000(MRL)	93.5 ± 4.3, 102.3 ± 10.1, 75.7 ± 20.2, 97.4 ± 128.1	4.6, 4.9, 6.7, 13.1
	β-cypermethrin	65.5	102.6	100, 200, 400, 1000(MRL)	77.9 ± 5.2, 99.9 ± 9.0, 97.2 ± 13.2, 89.9 ± 86.7	6.7, 4.5, 3.4, 9.6
	Cyfluthrin	68.6	97.3	100, 200, 400, 500(MRL)	82.9 ± 9.5, 89.9 ± 10.2, 108.5 ± 16.3, 94.8 ± 13.5	11.4, 5.7, 3.8, 2.9
	λ-cyhalothrin	72.1	104.3	100, 200, 400, 500(MRL)	88.0 ± 2.1, 95.8 ± 2.2, 102.5 ± 13.8, 87.9 ± 16.2	2.4, 1.1, 3.4, 3.7
	β-cyfluthrin,	72.7	102.1	100, 200, 400, 500(MRL)	92.0 ± 3.0, 83.5 ± 12.4, 79.1±19.8, 83.1 ± 33.0	3.3, 6.2, 6.3, 7.9
	Fenpropathrin	64.1	96.3	100, 200, 400, 1000(MRL)	80.7 ± 4.1, 75.2 ± 5.9, 72.6 ± 12.0, 93.5 ± 39.3	5.1, 3.9, 4.1, 4.2
	Deltamethrin	143.6	183.5	200, 400, 800, 2000	72.9 ± 14.6, 99.3 ± 26.2, 92.9 ± 40.1, 95.5 ± 112.9	13.4, 6.6, 5.4, 6.1

	Fenvalerate	148.4	185.5	200, 400, 800	98.8 ± 17.0, 97.5 ± 8.1, 93.0 ± 21.8	8.6, 2.1, 2.9
Leeks	Cypermethrin	72.2	99.5	100, 200, 400, 1000(MRL)	93.5 ± 4.3, 107.4 ± 16.9, 74.9 ± 19.3, 92.4 ± 97.2	4.6, 7.9, 6.5, 10.6
	β-cypermethrin	67.4	93.3	100, 200, 400, 1000(MRL)	78.7 ± 5.9, 112.4 ± 10.1, 79.9 ± 15.1, 86.8 ± 81.3	7.5, 4.5, 4.7, 9.4
	Cyfluthrin	64.1	95.6	100, 200, 400, 500(MRL)	108.2 ± 8.6, 99.6 ± 9.9, 65.1 ± 11.3, 96.1 ± 20.1	7.9, 5.0, 4.3, 4.2
	λ-cyhalothrin	75.8	105.2	100, 200, 400, 500(MRL)	87.6 ± 5.9, 96.4 ± 6.3, 100.5 ± 18.4, 85.9 ± 20.6	6.7, 3.2, 4.6, 10.8
	β-cyfluthrin,	67.3	100.9	100, 200, 400, 500(MRL)	106.2 ± 7.9, 73.7 ± 10.4, 74.2 ± 15.4, 73.1 ± 29.5	7.4, 7.0, 5.2, 8.1
	Fenpropathrin	65.6	97.1	100, 200, 400, 1000(MRL)	96.5 ± 8.6, 78.4 ± 4.0, 71.9 ± 8.3, 99.2 ± 20.1	8.9, 2.6, 2.9, 4.0
	Deltamethrin	152.2	188.6	200, 400, 800	92.3 ± 12.1, 97.9 ± 13.8, 93.3 ± 41.4	6.6, 3.5, 5.6
	Fenvalerate	142.1	181.5	200, 400, 800	103.6 ± 15.3, 105.7 ± 19.6, 94.6 ± 30.2	7.4, 4.6, 1.5

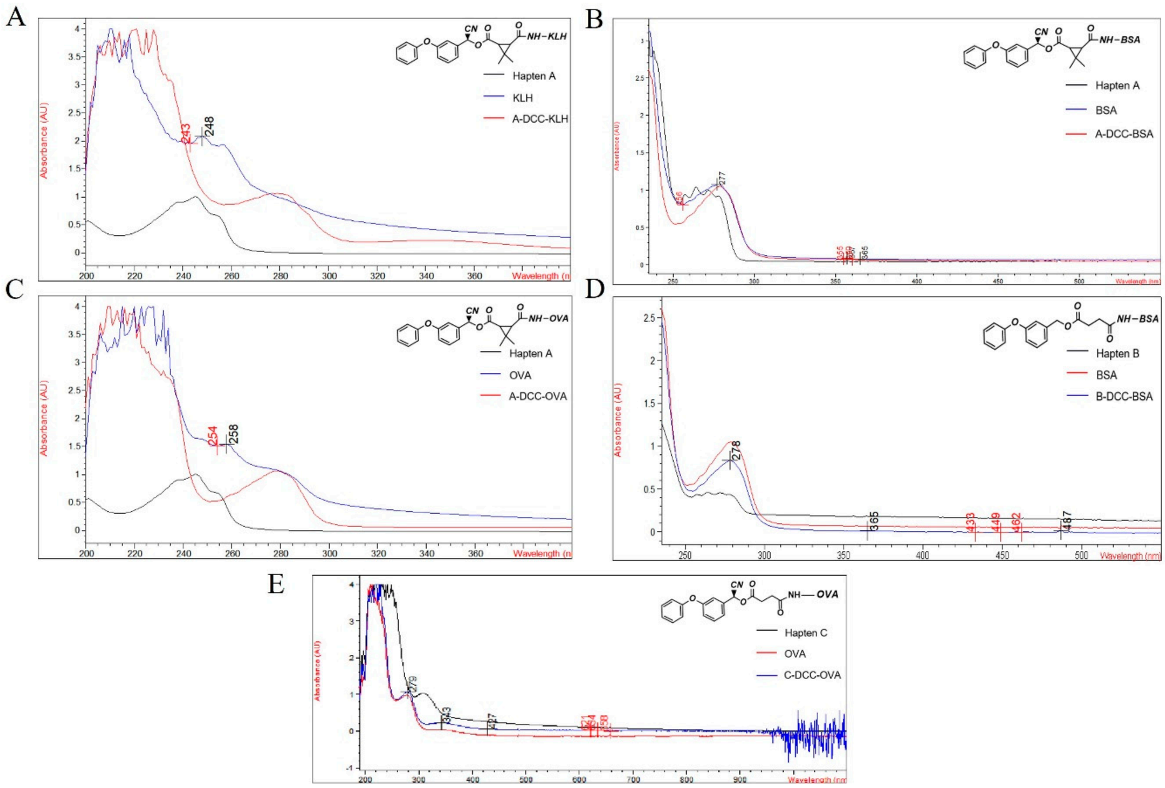

Figure S1. Ultraviolet absorption spectrums of A-DCC-KLH (A), A-DCC-BSA (B), A-DCC-OVA (C), B-DCC-BSA (D), C-DCC-OVA (E).

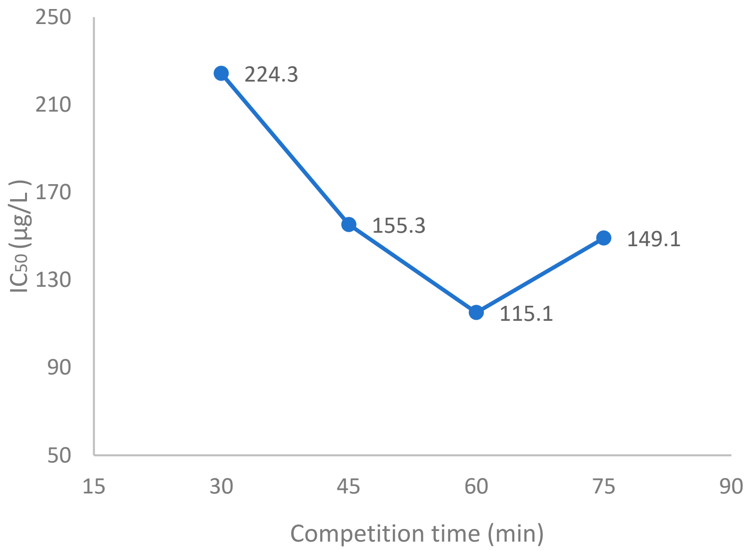

Figure S2. Screening of optimal competition time of ic-ELISA
